# Self-powered and broadband opto-sensor with bionic visual adaptation function based on multilayer γ-InSe flakes

**DOI:** 10.1038/s41377-023-01223-1

**Published:** 2023-07-24

**Authors:** Weizhen Liu, Xuhui Yang, Zhongqiang Wang, Yuanzheng Li, Jixiu Li, Qiushi Feng, Xiuhua Xie, Wei Xin, Haiyang Xu, Yichun Liu

**Affiliations:** 1 Key Laboratory of UV-Emitting Materials and Technology of Ministry of Education, Northeast Normal University, 130024 Changchun, China; 2 State Key Laboratory of Luminescence and Applications, Changchun Institute of Optics, Fine Mechanics and Physics, Chinese Academy of Sciences, No. 3888 Dongnanhu Road, Changchun, China

**Keywords:** Photonic devices, Optical sensors

## Abstract

Visual adaptation that can autonomously adjust the response to light stimuli is a basic function of artificial visual systems for intelligent bionic robots. To improve efficiency and reduce complexity, artificial visual systems with integrated visual adaptation functions based on a single device should be developed to replace traditional approaches that require complex circuitry and algorithms. Here, we have developed a single two-terminal opto-sensor based on multilayer γ-InSe flakes, which successfully emulated the visual adaptation behaviors with a new working mechanism combining the photo-pyroelectric and photo-thermoelectric effect. The device can operate in self-powered mode and exhibit good human-eye-like adaptation behaviors, which include broadband light-sensing image adaptation (from ultraviolet to near-infrared), near-complete photosensitivity recovery (99.6%), and synergetic visual adaptation, encouraging the advancement of intelligent opto-sensors and machine vision systems.

## Abstract (web summary)

A single two-terminal opto-sensor based on multilayer γ-InSe flakes was developed and successfully emulated human-eye-like adaptation behaviors, which could motivate the further development of advanced opto-sensors and artificial visual systems.

## Graphical abstract

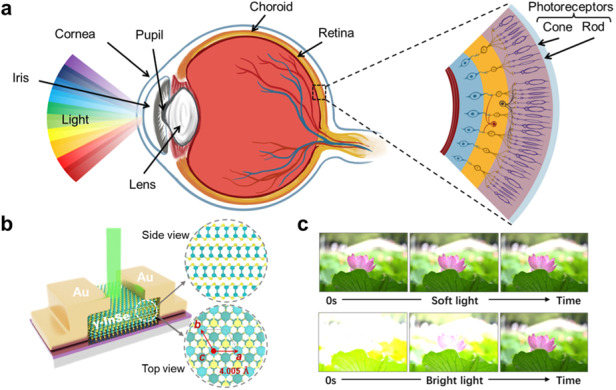

## Introduction

Visual perception, a vital sensing functionality for human beings and other vertebrates, contributes more than 80% of the perceptual information from the ambient environments to the brain^1,2^. With the rapid development of artificial intelligence, artificial visual systems are demanded to be able to mimic the visual perception capabilities of biological systems. Among them, an important functionality is visual adaptation, which can automatically adjust the response to stimuli according to different light environments^3,4^. For instance, the retina of the eye can reduce the levels of light dynamically to make the human-eye work normally when the illumination is from very dark to very bright. However, the existing efforts on mimicking visual adaptation functions have been trapped in complicated hardware and algorithms that typically reduce operating efficiency^5–8^. Ideally, a new generation of artificial visual systems with adaptation functions should have a simpler structure and lesser logic operations. Recently, several cutting-edge artificial visual systems based on a single device have been designed with built-in visual adaptation, such as organic transistor^9^, bilayer MoS_2_ phototransistor^10^, and heterojunction phototransistor^11^, which exhibit dynamic adaptation well to external light stimuli and show the potential to emulate human visual adaptation. However, their main mechanisms are still restricted to modulation of carrier trapping or ion migration, which are inadequate for the future development of visual adaptive devices and artificial visual systems. Hence, it is highly desired to explore more working mechanisms to serve the visual adaptation function of artificial visual systems that possess simple device architectures.

Visual adaptive devices based on pyroelectric materials may supply a new option for mimicking visual adaptation to constant light stimuli. That is because, upon light irradiation, the pyroelectric materials with noncentral-symmetric structure can convert time-dependent temperature fluctuation induced by the photothermal effect into instantaneous potential, which results from the changed polarization intensity with temperature causing interfacial charge release^12,13^. Such behavior of the instantaneous potential is similar to photosensitivity reduction over time in human vision systems when constant light is applied. It follows that pyroelectric materials show great possibilities in simulating human visual adaptation and need to be further explored. γ-InSe, as an emerging two-dimensional (2D) layered III−VI semiconductor, has typical low-symmetry crystal structures and belongs to *R3m* space groups^14–16^, similar to such typical pyroelectric materials as α-In_2_Se_3_, α-GeTe and GeSe^17–19^. More importantly, unlike transition-metal dichalcogenides (TMDs)^20,21^, γ-InSe does not require the construction of hybrid structures to enhance photon absorption because of its direct bandgap structure in multilayer regime^22^, which would undoubtedly simplify the device geometry. Together with ultrahigh mobility and broad spectral response^23–25^, multilayer γ-InSe is considered to have great potential in developing high-performance photoelectric conversion devices.

In this work, a two-terminal opto-sensor based on multilayer γ-InSe flake is experimentally demonstrated to emulate the human visual adaptation ranging from ultraviolet to near-infrared light without a bias voltage. When exposed to soft light, the device requires little adaptation process, but when the device is exposed from soft (or dim) to bright light, the response jumps initially and then declines to reach equilibrium, well simulating the self-adaptation process of human eyes to light stimuli. The main working mechanism for the dynamic adaptation is confirmed to be the synergy of photo-pyroelectric effect and photo-thermoelectric effect. Under bright light illumination, the photo-pyroelectric effect mainly causes the initial rapid adaptation process whereas the subsequent slow adaptation process is primarily induced by the photo-thermoelectric effect. Moreover, several important visual adaptation functions have been mimicked, including light-sensing image adaptation, photosensitive recoverability, and synergetic visual adaptation.

## Results

### Anatomy and visual adaptation of human eye

**Fig. 1:**
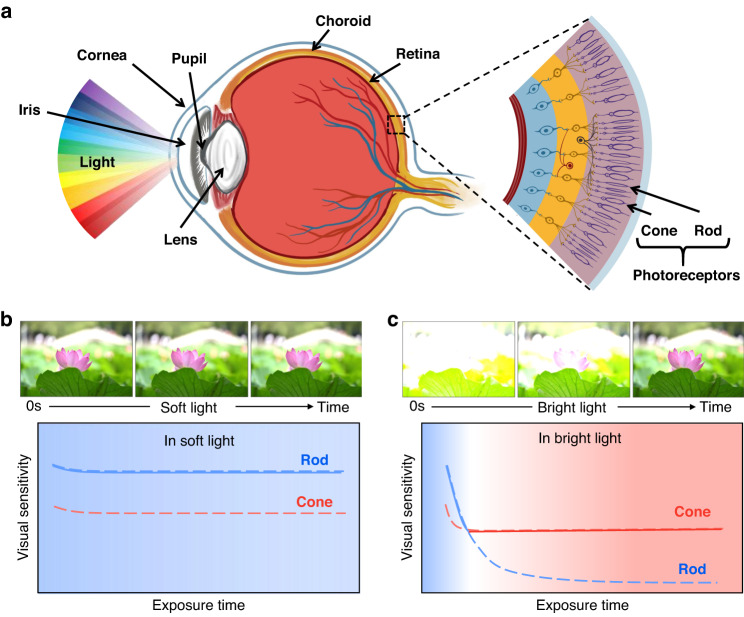
Visual adaptation of human eye. **a** Anatomy of human eye, mainly consisting of cornea, pupil, lens and vitreous body and retina. Cones and rods as photoreceptor cells in the retina are mainly responsible for perceiving the intensity of light, as well as the visual adaptation functions. Visual adaptation when exposed to **b** soft light and **c** bright light. Top: schematic diagram of how images of a lotus evolved through visual adaptation over time. Bottom: the mechanism of visual adaptation via dynamically switching between rod and cone cells

The human visual system, one of the most important organs of perception, mainly consists of a light refraction system, photoreceptor cells, the optic nerve, and the visual center. Here, the light emitted from external objects is firstly collected by photoreceptor cells (cones and rods) in the retina and then transformed into electrical signals that are transmitted to the brain to produce vision, as shown in Fig. 1a. Photoreceptor cells are mainly responsible for perceiving the intensity of light, as well as the visual adaptation functions^10,11^. Cones are more sensitive to bright light, while rods with high photosensitivity are experts at detecting dim light^26,27^. When always exposed to soft light, the sensitivity of the retina remains virtually unchanged so that the human-eye does not require a visual adaptation process and can consistently see objects clearly (Fig. 1b). Here, the switchover between cones and rods does not run but is in standby mode, which is essential for the human body to maintain low energy consumption. When exposed from soft (or dim) light to bright light, the human-eye will initially be dazzled by bright objects and progressively become able to see them clearly following visual adaptation (Fig. 1c). That is because the sensitivity of the retina gradually decreases over time, with rods and cones separately contributing to an initial rapid and a subsequent slow drop in sensitivity. Depending on the switchover between cone and rod cells through dynamic feedback, the human-eye could adapt and perceive a wide range of light illumination that is more than 160 dB^28^.

### Crystallographic structure characteristics of multilayer γ-InSe nanoflake

**Fig. 2:**
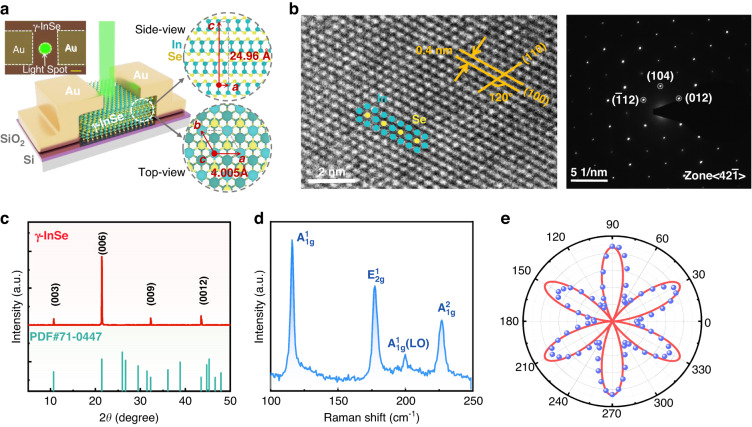
Crystallographic structure characteristics of multilayer γ-InSe nanoflake. **a** Schematic of the two-terminal multilayer γ-InSe-based opto-sensor. Inset: the optical microscopy image of the device. Scale bar: 20 μm. **b** Left panel: HR-TEM image of the multilayer γ-InSe nanoflake. Right panel: corresponding SAED image. **c** XRD pattern, **d** Raman spectra, and **e** angle dependence of SHG intensity and fitted curves of the prepared γ-InSe nanoflake

To simulate the visual adaptation of the human eye, we prepared a two-terminal opto-sensor based on multilayer γ-InSe flake that was mechanically exfoliated from the bulk materials, as shown in Fig. 2a. The optimized thickness of the γ-InSe flake (~240 nm) is recorded by atomic force microscope (AFM) (see Supplementary information Fig. S1). More opto-sensors with different thicknesses of γ-InSe flake are shown in Fig. S2. From the schematic side and top views of the γ-InSe flake, four atom sheets are covalently connected in each layer along the c-axis in the following order: Se-In-In-Se, forming a honeycomb-like In-Se lattice structure in the ab-plane. In a unit cell, the layered γ-InSe is made up of three layers that are stacked vertically and connected by weak Van der Waals force^29,30^. Meanwhile, the Se atoms of the second layer and the In atoms of the first layer are aligned with the In and Se atoms of the third layer, respectively. Depending on such stacking characteristics, γ-InSe exhibits low crystal-symmetry (*R3m* space group, similar to α-In_2_Se_3_, α-GeTe, GeSe, etc.^17–19^), which makes it the potential to be used as a pyroelectric material. Besides γ-phase, there are several crystallographic structures of InSe due to different symmetries and sequences of the stack, such as β-InSe and ε-InSe^31,32^. To ascertain the crystallographic structure of the multilayer InSe nanoflake, high-resolution transmission electron microscopy (HR-TEM) and the corresponding selected area electron diffraction (SAED) characterizations were performed (Fig. 2b). The HR-TEM image and SAED pattern separately display obvious crystal lattice fringes and clear diffraction spots with six-fold symmetry, indicating that the prepared multilayer nanoflake is of a high-quality monocrystalline crystal. From the HR-TEM image, inversion symmetry is absent from the atoms in the center of each smallest hexagon, consistent with the stacking mode of γ-InSe. In addition, the intersection angle of lattice fringes (marked in HR-TEM image) with 120 degrees matches the angle between the (100) and (010) planes of the hexagonal γ-InSe structure^33,34^, and the lattice spacing in the [100] direction and the d-spacing of the (100) plane are 0.4 nm and 0.2 nm, respectively. These data are well in line with previous results on the lattice constants of γ-InSe (*a* = *b* = 4.005 Å, *c* = 24.96 Å)^35,36^.

Subsequently, X-ray diffraction (XRD) and Raman spectroscopy are employed to further confirm the phase structures of the prepared InSe nanoflake. As can be seen, all diffraction peaks in the XRD patterns shown in Fig. 2c match well with the standard reference file (PDF71-0447) of γ-InSe, and no other peaks could be observed. The Raman spectrum of the InSe nanoflake is shown in Fig. 2d, and it contains three strong peaks at 113, 176, and 223 cm^-1^ as well as a weak peak at 199 cm^-1^, which are corresponding to A^1^_1 g_, E^1^_2 g_, A^2^_1 g_, and A^1^_1 g_(LO) modes, respectively^33,37^. Since the A^1^_1 g_(LO) mode is usually recognized as a significant characteristic for the non-centrosymmetric structure^36,38^, it can be unambiguously concluded that the structure of prepared InSe nanoflake is γ-phase, when paired with the aforementioned TEM and XRD results. Due to γ-InSe belonging to the *R3m* space group, such noncentral symmetry has the potential to produce strong second-harmonic generation (SHG), which is particularly sensitive to the broken inversion symmetry of the crystal. Therefore, polarization-dependent SHG is typically employed to confirm the crystallographic orientation and the symmetry property of γ-InSe nanoflake without causing any damage. In Fig. S3, the SHG peaks at 532 nm with diverse intensities are acquired as the incident polarized light direction varies, where the excitation source was a pulse laser at 1064 nm (see Experimental section for more details). As the incident polarized light direction is rotated from 0 to 355°, the polarization-dependent SHG intensity exhibits a clear six-lobe structure at various rotation angles due to the triple symmetry of the γ-InSe crystal structure (Fig. 2e), which is in good agreement with the previous SHG results of γ-phase InSe^35,39^.

### Light-intensity-dependent and broadband response characteristics of the γ-InSe opto-sensor

**Fig. 3:**
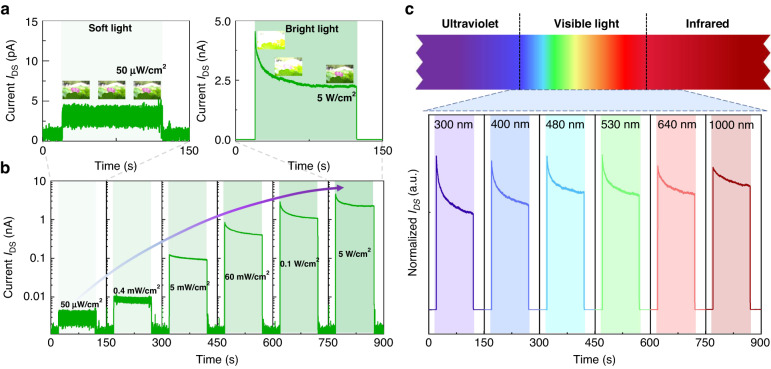
Light-intensity-dependent and broadband response characteristics of the γ-InSe opto-sensor. **a** Real-time current (*I*_DS_) of the device to various light stimuli, where a 532 nm CW laser with power densities of 50 μW/cm^2^ and 5 W/cm^2^ is employed to emulate the soft and bright light irradiation, respectively. **b** Power-dependent real-time *I*_DS_ on a semi-log scale, ranging from 50 μW/cm^2^ to 5 W/cm^2^. **c** Normalized real-time *I*_DS_ of the device under bright light irradiation (power density: 60 mW/cm^2^) at wavelengths between 300 and 1000 nm. All above the photo-responses are measured under zero bias voltage

As mentioned above, human visual adaptation requires not only a light-intensity-dependent transient response but also a dynamic adaptation to constant light stimuli. To this regard, we have measured the photocurrents of the prepared γ-InSe opto-sensor in which a 532 nm continuous-wave laser with power densities of 50 μW/cm^2^ and 5 W/cm^2^ is employed to emulate the soft and bright light irradiation, respectively. Here, all measurements of photocurrents are carried out under zero bias voltage, because the γ-InSe opto-sensor can act as a self-powered device, benefiting from the photocarrier separation induced by the built-in electric field at the Schottky junction interfaces between gold electrodes and multilayer γ-InSe. More analysis of the mechanisms will be discussed later. Intriguingly, the photocurrents to constant light stimuli exhibit obviously distinct behaviors over time for both irradiation conditions, as shown in Fig. 3a. Upon soft light (power density: 50 μW/cm^2^) irradiation, the photocurrent of the device always maintains a stable value below 5 pA, which is similar to the retinal sensitivity exposed to soft light, allowing for viewing objects clearly without visual adaptation process (Fig. 1b). When exposed to bright light (power density: 5 W/cm^2^), the device produces a dynamic photocurrent attenuation over time, initially rising to 4.5 nA after illumination and then decaying to 2.2 nA within a few tens of seconds to reach an equilibrium level. Such behavior is similar to how the retina automatically adjusts its sensitivity upon constant bright light irradiation, necessitating a period of adaptation before objects can be gradually seen (Fig. 1c).

Not only limited to these two light stimuli, the device also demonstrates real-time responses to light stimuli with varied power densities, ranging from 50 μW/cm^2^ to 5 W/cm^2^, as shown in Fig. 3b with a semi-log scale. With increasing the power densities, the photocurrents increase by three orders of magnitude, and a visible dynamic decay appears when the excitation power density exceeds 5 mW/cm^2^. It is analogous to the way that the sensitivity of the human-eye responds to constant stimuli with increasing light-intensity. Additionally, the multilayer γ-InSe can be employed for broad-band visual perception owing to its relatively small bandgap of ~1.2 eV, enabling the constructed device to perceive a wide range of light, from ultraviolet to near-infrared. Figure 3c shows the real-time current responses of the device under bright light irradiation (power density: 60 mW/cm^2^) at wavelengths between 300 and 1000 nm, in which all currents demonstrate obvious dynamic adaptation over time in self-powered mode. Furthermore, Fig. S4 demonstrates the light-intensity-dependent response curves excited at wavelengths between 300 and 1000 nm in self-powered mode. This broad-band light perception range of the γ-InSe opto-sensor even surpasses that of the human eye that is mainly restricted within the visible regime. Thus, it is reasonable to believe that the constructed γ-InSe opto-sensor, with low power-consumption and broadband light perception range, is a very promising building block for future artificial visual systems. Next, the corresponding working mechanism is analyzed in detailed to better comprehend the dynamic adaptation functions of the γ-InSe opto-sensor.

### Working mechanism of dynamic current decay

**Fig. 4:**
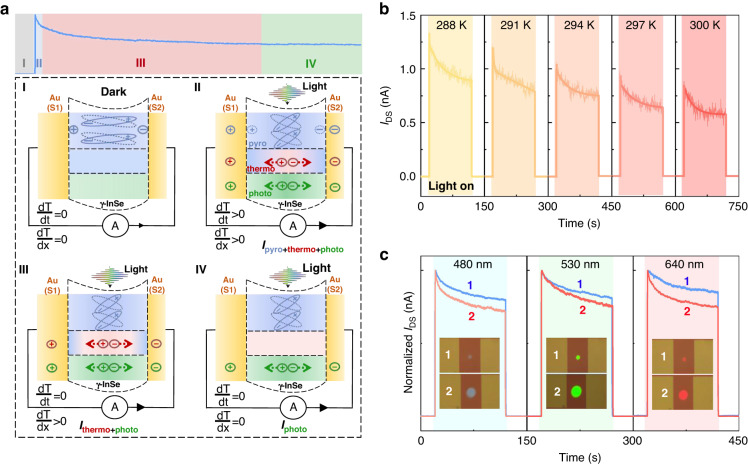
Working mechanism of dynamic current decay in the γ-InSe device. **a** Top: an enlarged adaptation curve upon 532 nm laser irradiation that can be divided into four stages (I, II, III, and IV). Bottom: schematic illustrations of dynamic current decay, corresponding to the four stages denoted in top panel. **b** Temperature-dependent real-time *I*_DS_ upon 532 nm laser irradiation (power intensity: 60 mW/cm^2^) from 288 to 300 K at zero bias and without cooling. **c** Normalized real-time *I*_DS_ excited by 480 nm, 530 nm, 640 nm light at the same power density of 60 mW/cm^2^ with different areas of light illumination under zero bias voltage, including a small illumination area of about 60 μm^2^ (labeled as 1) and a large one of about 360 μm^2^ (labeled as 2)

Figure 4a top panel depicts a typical enlarged adaptation curve upon 532 nm laser irradiation and it can be separated into four main stages, including (I) an initial state before the light stimulus, (II) a peak state shortly after the light stimulus, (III) a subsequent gradual adaptation state, and (IV) reaching a new equilibrium state. Here, we propose that the synergy of photo-pyroelectric effect and photo-thermoelectric effect together contributes to the main operating mechanism of visual adaptation in the γ-InSe opto-sensor (Fig. 4a, bottom). Without light stimulus, the main response of the device in the (I) stage is a weak dark current, which is equivalent to a closed-eye state. It is well known that the photothermal effect, which converts light into heat and is enhanced with the increasing intensity of light irradiation, occurs ineluctably when a device is exposed to light^40–43^. Accompanied by heat generated at the light spot due to the photothermal effect, the γ-InSe device will experience a transient temperature increase (dT/dt > 0) and a spatial temperature gradient (dT/dx > 0). As a result, the pyroelectric effect, that is, the charge release phenomenon induced by the change in polarization intensity with temperature variations^12^, and thermoelectric effect, that is, the temperature difference between pair of gold electrodes in asymmetric contact with diverse Seebeck coefficients (see Fig. S5) is directly converted to electric voltage^44^, may be able to co-contribute to the currents of the device during light illumination. Additionally, the photovoltaic effect that separates photocarriers by built-in electric field at the Schottky junction interfaces between gold electrodes and multilayer γ-InSe, also contributes to the overall output currents. In the (II) stage, therefore, a sharp output current after the light stimulus mainly contains three parts of contribution from the photo-pyroelectric effect, photo-thermoelectric effect, and photovoltaic effect. For more clarity, the corresponding output current is denoted as *I*_pyro+thermo+photo_.

Subsequently, the output current shows a slow decay after rapidly decreasing in the (III) stage, because the temperature tending to constant (dT/dt = 0) impels the pyroelectric potential to disappear rapidly, leaving the current contributions from the photo-thermoelectric effect and photovoltaic effect. Accordingly, the output current is denoted as *I*_thermo+photo_. In the (IV) stage, the output current decreases to a steady value because the device temperature reaches a new thermal equilibrium, eliminating the spatial temperature gradient (dT/dx = 0) and minimizing the photo-thermoelectric effect. Hence, the output current at this stage is merely from the contribution of photovoltaic effect (denoted as *I*_photo_). It follows that the synergy of photo-pyroelectric effect and photo-thermoelectric effect should be mainly responsible for the dynamic adaptation functionality of the device when exposed to bright light. In contrast, the photothermal effect under soft (or dim) light irradiation (less than 5 mW/cm^2^) generates too little heat for γ-InSe flake to induce obvious change in transient temperature increase and spatial temperature gradient. As a result, both the photo-pyroelectric and photo-thermoelectric effect would be significantly suppressed, and therefore the currents exhibit a steady value over time (Fig. 3a), corresponding to the (IV) stage.

To prove the above hypothesis, firstly, temperature-dependent real-time current measurements were performed upon 532 nm laser illumination (power density: 60 mW/cm^2^). Figure 4b demonstrates that the current response is obviously dependent on the measurement temperature. With increasing temperature from 288 K to 300 K, the output current that is mostly concentrated in the (II) and (III) stages gradually decreases, demonstrating the dynamic decay of current comes from the photo-pyroelectric effect and photo-thermoelectric effect^45^. This is because, under the same lighting conditions, a higher background temperature would result in a slower temperature-change rate (dT/dt) and a smaller spatial temperature gradient (dT/dx), which is bound to limit the currents contributed by the photo-pyroelectric effect and photo-thermoelectric effect. According to previous reports on pyroelectric materials^46,47^, as the light illumination is turned on (off), the sharp rising (falling) edge of output current is indeed induced by the pyroelectric effect as a result of an instantaneous temperature increase (decrease), which agrees well with our observations (Fig. S6). Hence, it can be concluded that the sharp output current after the light stimulus in the (II) stage is mainly contributed by the photo-pyroelectric effect. Subsequently, we measured the real-time currents with different illumination areas at the same power density of 60 mW/cm^2^. Since the larger irradiation area would result in a shorter time of reaching thermal equilibrium, therefore, the output current contributed by the photo-thermoelectric effect for larger irradiation area would demonstrate a faster decay in the (III) stage than that of smaller irradiation area, which is experimentally verified by different irradiation wavelengths, including 480, 530, and 640 nm (Fig. 4c). In terms of timescale, previous work has reported that the fast process within several seconds is contributed by photo-pyroelectric effect while the slow process within tens of seconds is induced by photo-thermoelectric effect^45^, consistent with our fitting results of dynamic current decay (see Fig. S7). As for the photovoltaic effect, it can be well validated by the *I*_DS_–*V*_DS_ characteristic curve of the device under light illumination (see Fig. S8), which exhibits an obvious current rectification behavior and is in common with the behaviors of built-in electric field^48,49^. Moreover, the dynamic decay of currents induced by carrier trapping from interfaces between the γ-InSe and substrate is ruled out in our case (Fig. S9). To sum up, the dynamic decay of currents over time indeed arises from the synergy of photo-pyroelectric effect and photo-thermoelectric effect, which can be well applied to the simulation of photoadaptation function of artificial visual devices. Compared with previous works that used carrier trapping to dynamically control the response to light stimuli^9,10,50^, the constructed γ-InSe opto-sensor can operate in self-powered mode and exhibit a wide wavelength response range, which are very important for the further development of advanced artificial visual systems with low power-consumption and broadband wavelength perception range. Here, the characteristics of the constructed γ-InSe opto-sensor and that reported in other visual adaptive devices were compared and summarized in Table 1.

**Table 1:** Comparing the characteristics of the γ-InSe opto-sensor in this work to visual adaptive devices in previous works

Materials	Device type	Self-powered mode	Spectral response range	Photopic adaptation	Scotopic adaptation	Ref.
CsFAMA	Two-terminal	×	Vis–NIR	√	×	^2^
P3HT:PCBM	Three-terminal	×	Vis	√	×	^9^
Bilayer MoS_2_	Three-terminal	×	Vis	√	√	^10^
CsPb(Br_1-x_I_x_)_3_/MoS_2_	Three-terminal	×	UV–Vis	√	×	^11^
CsPbBr_3_/MoS_2_	Three-terminal	×	UV	√	×	^50^
γ-InSe	Two-terminal	√	UV-NIR	√	×	This work

### Realization of visual adaptation functions by the γ-InSe opto-sensor

**Fig. 5:**
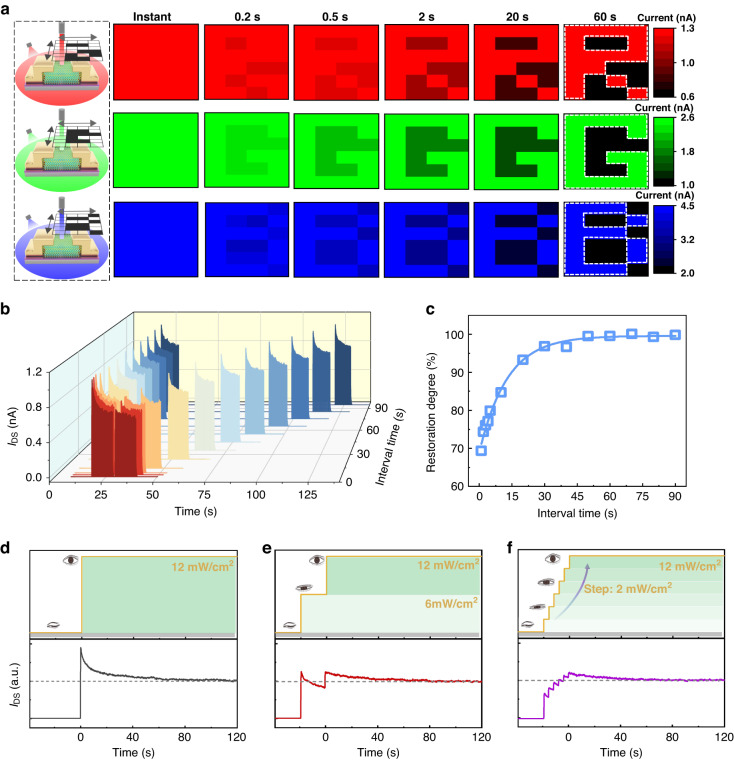
Bioinspired visual adaptation functions of the γ-InSe opto-sensor. **a** Left: schematic of the equipment of 2D movable hollow mask. Right: light-sensing image adaptation of letter “R”, “G”, and “B” pattern irradiated by 638 nm (red light), 532 nm (green light), and 405 nm (blue light) laser illumination (200 mW/cm^2^) under a bright background (100 mW/cm^2^), respectively. **b** Real-time *I*_DS_ triggered by two bright light stimuli with various interval time. **c** The restoration degree as a function of interval time. With the interval time increasing to 60 s, the restoration degree reaches a near-unity value of 99.6%. Mimicking the synergetic visual adaptation with eyelid in three different scenarios to bright light stimuli (12 mW/cm^2^), including **d** opening the eye directly, **e** first-half-opening and then fully opening the eye, and **f** slowly opening the eye in six stages. All above the measurements are carried out under zero bias voltage

Next, several important visual adaptation functions in the human retina have been mimicked with the γ-InSe opto-sensor. Based on the broad-band response characteristic of the device, we have emulated the light-sensing image dynamic adaptation at 638 nm, 532 nm, and 405 nm laser illumination, corresponding to red (R), green (G), and blue (B) light, respectively. Here, we separately put a movable hollow mask (6 × 4 pixels) with the letter “R”, “G”, and “B” pattern in front of the three light sources to achieve the image sensing (Fig. 5a), where the light can pass through the vacant pixels and is blocked by the solid pixels. As a result, the γ-InSe device is exposed to a strong light of 200 mW/cm^2^ when the pixels of the pattern of “R”, “G”, and “B” are moved to the fixed light source, but for other pixels, the device is only exposed to a bright background illumination (100 mW/cm^2^). When exposed to red light, we define *I*_DS_ = 0.6 nA as the gray level of 0 and *I*_DS_ = 1.3 nA as the gray level of 255 to maintain uniformity of perception in the receptive field, because the ganglion cell outputs in the biological visual system actually fall within a fixed range despite the wide range of light-intensity^10^. In the case of green light, the two values are separately 1.0 nA and 2.6 nA, whereas 2.0 nA and 4.5 nA apply to blue light. Note that the gray level value of the device is 255 as a result of saturation when subjected to three bright lights of 200 mW/cm^2^. The perceived patterns of “R”, “G”, and “B” at 0.2, 0.5, 2, 20, and 60 s are displayed in Fig. 5a, which are taken from respective *I*_DS_ (more details are shown in Fig. S10). The initial “dazzling” pattern of “R”, “G”, and “B” gradually evolves into a clear image over time, similar to the visual adaptation process of the retina. Interestingly, the demonstrated speed of light-sensing image adaptation is also on par with the human visual system^51,52^.

The recoverability of retinal photosensitivity, which is defined as returning to initial retinal sensitivity following visual adaptation, is also an essential for the functioning of the human visual system. To assess the recoverability of the γ-InSe device, we have examined the current response of the device triggered by two light stimuli with various interval time (Fig. 5b). When the interval time is set to 1 s, the subsequent stimulus generates a substantially lower peak current of 0.7 nA as compared to the peak current response of 1.1 nA triggered by the first stimulus. As the interval time in darkness increases, the responses gradually recover on a timescale of about 60 s. The recoverability can be quantitatively evaluated by using the ratio of peak current triggered by post-stimulus to that triggered by pre-stimulus (*I*_post-peak_/*I*_pre-peak_), which is defined as restoration degree. Figure 5c shows the restoration degree as a function of interval time. As the interval time is increased, the restoration degree gradually improves, reaching a near-unity value of 99.6% at the interval time of 60 s. This is because enough interval time in darkness could render the temperature of the device down by re-establishing thermal equilibrium with the surrounding environment. When exposed to the bright light stimulus again, the γ-InSe device needs to re-experience the photoadaptation process, just similar to the re-adaptation process of human eye after staying in darkness for a period of time.

Finally, the collaborative process of retina with eyelid, another important function of human visual system, has also been successfully simulated by our γ-InSe opto-sensor. In reality, the retina is not the only apparatus of human-eye participating in visual adaptation. The eyelid and pupil could dynamically control the amount of light flux entering the human eye, assisting the retina in finishing the process of visual adaptation. For convenience, we simplify the process of controlling the light flux into the human eye and only consider the role of the eyelid. Here, three different scenarios to constant bright light stimuli (power density: 12 mW/cm^2^) were simulated, namely, opening the eye directly, first-half-opening and then fully opening the eye, and slowly opening the eye in six stages. In the first scenario, the eyelid is not participating in the visual adaptation process and only the retina is active, resulting in a longer adaptation time (~70 s) to reach the equilibrium level (Fig. 5d). When the eye is first-half-opened and then fully opened, the light flux into the human eye is dynamically controlled by the eyelid and the adaptation time of reaching the equilibrium level is significantly reduced to ~50 s (Fig. 5e). For the third scenario, since the eyelids are more actively involved in the visual adaptation process, the corresponding adaptation time is further shortened to ~40 s (Fig. 5f). When the light flux entering the retina is effectively adjusted, the simulated adaptation time gradually decreases, coinciding well with the actual situation. That is, the eyelid and pupil cooperate to dynamically control the light flux entering the human eye, enabling the retina to more effectively and swiftly adapt to constant bright light stimuli. Corresponding to the device, such a progressive increase in light-intensity slows the rate of temperature rise (dT/dt) so that the currents contributed by the photo-pyroelectric effect are markedly reduced, resulting in the total currents decaying more quickly to a constant value. These results, including light-sensing image dynamic adaptation, photosensitive recoverability, and synergetic adaptation with eyelid, indicate that the constructed γ-InSe opto-sensor could be considered as a promising functional component for future advanced artificial visual systems.

## Discussion

In summary, we have developed a single two-terminal opto-sensor based on multilayer γ-InSe flakes, which well simulated the visual adaptation behaviors. The new working mechanism of combining of the photo-pyroelectric effect and photo-thermoelectric effect enables the dynamic adaptation of device currents in response to constant light stimulus. Benefiting from the relatively small bandgap of γ-InSe and the photovoltaic effect, the device can perceive a wide range of light from ultraviolet to near-infrared regime and operate in self-powered mode. Most importantly, the constructed device exhibits good human-eye-like adaptation behaviors, including broadband light-sensing image adaptation, near-unity photosensitivity recoverability, and synergetic visual adaptation function. Our studies not only could enrich the working mechanism of simulating human visual adaptation, but also may motivate the further development of advanced opto-sensors and artificial visual systems.

## Materials and methods

### Fabrication of γ-InSe opto-sensor

Firstly, the multilayer γ-InSe nanoflake that was mechanically exfoliated from bulk crystals was transferred by PDMS film with a thickness of 500 μm onto a clean 300 nm SiO_2_/Si substrate. Using a TEM copper grid as the mask, the patterned gold electrodes were fabricated by typical vacuum-thermal-evaporation method on a sacrifice substrate (soda-lime glass) that had been treated with oxygen plasma for 15 min before being soaked in octadecyl trichlorosilane to make the surface hydrophobic. Subsequently, the patterned Au electrodes on the soda-lime glass were transferred onto the exfoliated γ-InSe flakes accurately with the adhesion of PVA/PDMS composite film using a self-made high-precision transfer system. Relying on the irregular shape of the exfoliated multilayer γ-InSe flake, a pair of electrodes in asymmetric contacts can be easily obtained (Fig. S3), accompanied by diverse Seebeck coefficients. Lastly, the PVA layer can be dissolved via dipping in the ultrapure water for 4 h, and the PDMS layer is released by heating to 373 K. To optimize the interfacial contacts between electrodes and γ-InSe, the device was annealed under vacuum at 423 K for 10 min.

### Characterization of multilayer γ-InSe

The thickness of the γ-InSe flakes is characterized by AFM (Dimension Icon, Bruker) in the tapping mode. The HR-TEM and the SAED pattern are done with a JEOL JEM 2100 F system operated with an acceleration voltage of 200 kV. The XRD (Rigaku D/max-2500) and Raman spectroscopy (Horiba, HR Evolution) are performed to further confirm the phase of the γ-InSe flakes. For the SHG measurements, the excitation source is a pulse laser with pulse width of 10 ps, repetition rate of 50 MHz and output energy of ~1.16 eV (1064 nm), and focused on sample by a 100× objective with 0.9 numerical aperture (NA). The polarization-dependent SHG are gathered by rotating the half-wave plate with a step of 5 degrees relative to a fixed light polarization.

### Characterization of device

All electrical and photoelectric characteristics of the device were measured with a commercial integrated platform, which is equipped with a source-meter (Keithley, 2614b), several CW lasers (405 nm, 532 nm, 638 nm), a xenon lamp (300–1100 nm) and a monochromator and so forth. The temperature-dependent real-time currents were recorded with the help of a temperature-controlled stage (Linkam, THMS350V).

## Supplementary information

Supplementary information for Self-powered and Broadband Opto-sensor with Bionic Visual Adaptation Function Based on Multilayer γ-InSe Flakes

## Data Availability

Data is available from corresponding author upon reasonable requirements.
