# Supplementary material for: Self-powered and broadband opto-sensor with bionic visual adaptation function based on multilayer γ-InSe flakes
Source: Light Sci Appl. 2023 Jul 24;12:180. doi: 10.1038/s41377-023-01223-1 (PMC10366227; doi:10.1038/s41377-023-01223-1)
Supplement: Supplementary file 1 — Supplementary information for Self-powered and Broadband Opto-sensor with Bionic Visual Adaptation Function Based on Multilayer γ-InSe Flakes [file 41377_2023_1223_MOESM1_ESM.docx]

Supplementary information for

**Self-powered and Broadband Opto-sensor with Bionic Visual Adaptation Function Based on Multilayer γ-InSe Flakes**

Weizhen Liu,^1^ Xuhui Yang,^1^ Zhongqiang Wang,^1^ Yuanzheng Li,^1,^* Jixiu Li,^1^ Qiushi Feng,^1^Xiuhua Xie,^2^Wei Xin,^1^ Haiyang Xu^1,^* and Yichun Liu^1^

^1^Key Laboratory of UV-Emitting Materials and Technology of Ministry of Education, Northeast Normal University, Changchun 130024, China

^2^State Key Laboratory of Luminescence and Applications, Changchun Institute of Optics, Fine Mechanics and Physics, Chinese Academy of Sciences, No. 3888 Dongnanhu Road, Changchun, China

*Corresponding authors: liyz264@nenu.edu.cn and hyxu@nenu.edu.cn

**Figure S1.** (a) AFM image of the multilayer γ-InSe. The dashed white line represents the scan line, which spans across the gold electrode, multilayer γ-InSe flake and substrate regions. (b) Corresponding height profile shows the thickness of electrode is ~20 nm and that of the γ-InSe flake is ~240 nm.

**Figure S2.** Light-intensity-dependent real-time *I_DS_* measurements for the opto-sensors with different thicknesses of γ-InSe flake, including (a) ~2.87 μm, (b) ~356.3 nm, (c) ~139.1 nm and (d) ~36.7 nm. All opto-sensors operate in self-powered mode under 532 nm laser excitation.

With the gradual thinning of thickness of γ-InSe flake, the threshold power density of excitation light that generates dynamic photocurrent decay gradually decreases. When the thickness of γ-InSe flake is beyond ~500 nm, the real-time photocurrent curves almost do not exhibit dynamic decay over time at any given light power density (Figure S2a). This is because for thicker γ-InSe flake, the heat generated at the light spot due to photothermal effect, can only induce a lesser change in transient temperature increase and spatial temperature gradient for the γ-InSe device. Hence, the photo-pyroelectric and photo-thermoelectric effect are relatively weaker in the opto-sensor with thicker γ-InSe flake, exhibiting an unobvious dynamic current decay. For thinner γ-InSe flake (Figure S2b and S2c), the heat generated at the light spot would induce a larger change in transient temperature increase and spatial temperature gradient, leading to enhanced photo-pyroelectric and photo-thermoelectric effect.

However, the thickness of γ-InSe is not the thinner the better for the performance of opto-sensor. When the thickness of γ-InSe flake is lower than ~50 nm, although dynamic current decay over time can be still observed (Figure S2d), the current response drops by two orders of magnitude, due to reduced light absorption. To sum up, we prepared the opto-sensor using an optimized thickness of γ-InSe (~200 nm) to gain a good device performance (Figure 2).

**Figure S3.** The SHG spectra of γ-InSe nanoflake at different incident polarized light directions from 0° to 60°. As the angle increases from 0 to 60°, the SHG intensity at 532 nm initially rises and then falls, where the excitation source is a picosecond pulse laser at 1064 nm. Due to the six-fold rotational symmetry of γ-InSe, only one repetition cycle of SHG spectra is selected to be displayed here.

**Figure S4.** Light-intensity-dependent adaptive curve of the γ-InSe opto-sensor at wavelengths between 300 and 1000 nm. Here, all current responses exhibit visible dynamic decay over time in self-powered mode, when the excitation power density exceeds 0.5 mW/cm^2^. It is analogous to the way that the sensitivity of the human eye responds to constant bright light stimuli.

**Figure S5.** The optical microscopy image of a pair of gold electrodes in asymmetric contacts with γ-InSe. Relying on the irregular shape of the γ-InSe flake, a pair of electrodes in asymmetric contacts can be easily obtained. The prepared pair of gold electrodes in asymmetric contacts can cause diverse Seebeck coefficients and Schottky junction interfaces.

**Figure S6.** Real-time currents irradiated by 532 nm light under 0 V, -0.01 V, -0.03 V, -0.05 V bias voltage.

As the light illumination is turned on and off, the pyroelectric effect could separately induce forward current spike and reverse current spike due to transient temperature variations. However, the reverse current spike is not obvious in contrast to the forward spike under zero bias voltage, which is similar to previous works.^1-3^ This could be because the reverse current spike induced by changed polarization intensity with temperature is suppressed due to the existence of the forward photovoltaic field. As the increased reverse bias is applied from -0.01 V to -0.05 V, the forward photovoltaic field is gradually weakened so that the reversed spike is gradually obvious, confirming the existence of pyroelectric effect in the γ-InSe flake.

**Figure S7.** (a) Typical real-time *I_DS_* of the γ-InSe opto-sensor upon 532 nm laser (power density=100 mW/cm^2^) irradiation. (b) Fitting result of the dynamic current decay shown in (a).

The dynamic current decay that can be well fitted with a double exponential decay function contains a fast process of ~3.2 s and a slow process of ~34.1 s. According to the previous report,^4^ the dynamic current decay exactly exhibits two different processes for the working mechanism of the device based on the coupled effect of photo-pyroelectric and photo-thermoelectric effect. The fast process within several seconds is mainly contributed by the photo-pyroelectric effect while the slow process within tens of seconds is induced by the photo-thermoelectric effect, consistent with our fitting results.

For the visual adaptation of human eye, there are also two different decay processes in the sensitivity of the retina during visual adaptation. Here, the rods are mainly responsible for the fast decay process of the sensitivity of the retina, whereas the cones are mainly responsible for the slow decay process.^5,6^ Correspondingly, the two decay processes of the sensitivity of the retina basically match our fitting results in timescale, which is very meaningful for simulating human visual adaptation.

**Figure S8**. *I*_DS_-*V*_DS_ characteristics under 532 nm laser irradiation with different output powers. It can be seen that the open-circuit voltage (V_oc_) gradually rises as the output power increases from 5 mW/cm^2^ to 5 W/cm^2^. The V_oc_ can reach up to 0.71 V and a significant short-circuit current (~3 × 10^-8^ A) appears at a power density of 5 W/cm^2^, indicating a remarkable photovoltaic effect.

**Figure S9.** (a) Left panel: the schematic of the two-terminal opto-sensor with a vertical structure of Au/γ-InSe/h-BN/SiO_2_/Si. Right panel: corresponding optical microscopy image of the device. Scale bar: 20 μm. (b) Power-dependent real-time *I*_DS_ on a semi-log scale of the device, ranging from 50 μW/cm^2^ to 5 W/cm^2^. The excitation source is a 532 nm CW laser.

Referring to previous work,^7^ h-BN with surface free of charged impurities and dangling bonds has been widely used as an ideal atomically flat substrate and insulating layers for many two-dimensional layered materials. To rule out the carrier trapping from interfaces between the γ-InSe and substrate, therefore, we have chosen the h-BN flake that was mechanically exfoliated from the high-quality bulk materials to replace SiO_2_/Si as the substrate of the device. The corresponding optical microscopy image is shown in Figure S9a. Intriguingly, the power-dependent real-time current of the device (Figure S9b) demonstrates the same trend with that shown in Figure 3b, and an obvious dynamic decay of current can be observed upon bright light illumination (more than 5 mW/cm^2^). Hence, it is possible to rule out the dynamic decay of currents that is caused by the carrier trapping from interfaces between the γ-InSe and substrate in our scenario.

**Figure S10.** Top of (a), (b) and (c) show the schematic diagram of 2D movable hollow masks of letter “R”, “G” and “B” pattern (6 × 4 pixels), respectively. Taking the “R” pattern as an example, the light source is a 638 nm CW laser and can pass through the 16 vacant pixels (corresponding to “signal”), but for other 8 solid pixels (corresponding to “background”), the light is blocked. As a result, the γ-InSe device would be exposed to a strong light of 200 mW/cm^2^ when the pixels of the pattern of “R”, “G”, and “B” are moved to the fixed light source, but the device is only exposed to a bright background illumination (100 mW/cm^2^) for other pixels. Bottom of (a), (b) and (c) demonstrate the time-dependent *I*_DS_ of the device at zero bias voltage in pixels of “signal” and “background” upon red (638 nm), green (532 nm), and blue (405 nm) light irradiation, respectively.

**References:**

1. Liu, Y. *et al.* Enhanced photocurrent in ferroelectric Bi_0. 5_Na_0. 5_TiO_3_ materials via ferro-pyro-phototronic effect. *Nano Energy* **98**, 107312 (2022).

2. Zhang, Y. *et al.* Highly Sensitive Photoelectric Detection and Imaging Enhanced by the Pyro‐Phototronic Effect Based on a Photoinduced Dynamic Schottky Effect in 4H‐SiC. *Advanced Materials* **34**, 2204363 (2022).

3. Wang, D. *et al.* Self-powered ZnO/SrCoOx flexible ultraviolet detectors processed at room temperature. *Materials & Design* **203**, 109616 (2021).

4. Liu, S. *et al.* A bioinspired broadband self-powered photodetector based on photo-pyroelectric-thermoelectric effect able to detect human radiation. *Nano Energy* **93**, 106812 (2022).

5. Baccus, S. A. *et al.* Fast and slow contrast adaptation in retinal circuitry. *Neuron* **36**, 909-919 (2002).

6. Martinez Canada *et al.* *13th International Work-Conference on Artificial Neural Networks (IWANN).* 175-184.

7. Moon, S. *et al.* Hexagonal Boron Nitride for Next‐Generation Photonics and Electronics. *Advanced Materials* **35**, 2204161 (2022).
